# Early prediction of gallstone disease with a machine learning-based method from bioimpedance and laboratory data

**DOI:** 10.1097/MD.0000000000037258

**Published:** 2024-02-23

**Authors:** İrfan Esen, Hilal Arslan, Selin Aktürk Esen, Mervenur Gülşen, Nimet Kültekin, Oğuzhan Özdemir

**Affiliations:** a Yüksek İhtisas University, Faculty of Medicine Department of Internal Medicine, Ankara, Turkey; b Ankara Yildirim Beyazit University, Department of Software Engineering, Faculty of Engineering and Natural Sciences, Ankara, Turkey; c Ankara City Hospital, Medical Oncology Clinic, Ankara, Turkey; d Keçiören VM Medicalpark Hospital, Department of Nutrition and Dietetics, Ankara, Turkey; e Yüksek İhtisas University, Faculty of Medicine Department of Department of Radiology, Ankara, Turkey.

**Keywords:** bioimpedance, gallstones, laboratory data, machine learning, prediction

## Abstract

Gallstone disease (GD) is a common gastrointestinal disease. Although traditional diagnostic techniques, such as ultrasonography, CT, and MRI, detect gallstones, they have some limitations, including high cost and potential inaccuracies in certain populations. This study proposes a machine learning-based prediction model for gallstone disease using bioimpedance and laboratory data. A dataset of 319 samples, comprising161 gallstone patients and 158 healthy controls, was curated. The dataset comprised 38 attributes of the participants, including age, weight, height, blood test results, and bioimpedance data, and it contributed to the literature on gallstones as a new dataset. State-of-the-art machine learning techniques were performed on the dataset to detect gallstones. The experimental results showed that vitamin D, C-reactive protein (CRP) level, total body water, and lean mass are crucial features, and the gradient boosting technique achieved the highest accuracy (85.42%) in predicting gallstones. The proposed technique offers a viable alternative to conventional imaging techniques for early prediction of gallstone disease.

## 1. Introduction

Gallstone disease is a leading concern among digestive system diseases worldwide, with a 10% to 20% prevalence among European adults. The occurrence of the disease is continuously rising_,_^[1–3]^ and the factors leading to gallstone formation are multifaceted, ranging from sex-specific, genetic, and lifestyle factors to other health conditions. The disease can range from asymptomatic carriers to symptomatic forms such as acute cholecystitis and bile duct stones.^[4]^ Research published by Ogiela et al^[5]^ indicated that there is a strong correlation between gallstone disease and insulin resistance, systemic inflammation, and genetic predispositions. Another study showed that cholesterol is a major component of 75% to 80% of gallstones found in Western countries and is often associated with systemic abnormalities.^[6]^ Factors such as age and sex play a role in gallstone risk, with women being at a higher risk due to childbirth and hormonal cycles_._^[7–10]^ The rising incidence of obesity and metabolic syndrome is correlated with an increase in gallstone formation.^[11–13]^ This relationship was further confirmed by the involvement of insulin resistance and systemic inflammation.^[14]^ Transabdominal ultrasonography remains the preferred detection tool for gallstones, with a sensitivity of 84% and a specificity of 99%.^[15–17]^ However, its efficacy can be compromised under certain conditions, particularly for stones smaller than 3 mm.^[18,19]^

Bioimpedance Analysis (BIA)^[20]^ is a noninvasive and cost-effective method that is extensively used for body composition measurements, including fat mass, lean mass, and total body water. It is also effective in detecting gallstones.^[21,22]^ BIA operates on the principle that the resistance of electrical flow (impedance) differs across various body tissues, allowing quantifiable measurements that are indicative of a person’s health status.^[23]^ Metabolic markers, routinely gauged in clinical laboratories, offer insight into an individual’s metabolic health. These markers, which encompass parameters such as cholesterol, triglyceride, and glucose levels, serve as harbors for various disorders when present in abnormal concentrations. Aberrations in certain metabolic markers are implicated in gallstone formation.^[24]^ Recent explorations suggest a noteworthy correlation between measurements derived from BIA and these pivotal metabolic markers.^[25]^ By extending this association, BIA might serve as an early detector for metabolic disruptions that predispose individuals to gallstones. Given its noninvasive nature, BIA is a pivotal tool in early screening, enabling the timely identification of individuals at higher risk. Although direct links between BIA measurements and gallstone risk have been sparse in the literature, emerging research has underscored the potential correlation between BIA-derived data and metabolic markers.^[26]^ For instance, some studies have highlighted the relationship between altered body composition and dyslipidemia, which is a prominent risk factor for gallstones.^[27,28]^ Identifying at-risk individuals at risk for gallstone diseases is of paramount importance, allowing timely clinical interventions and minimizing complications.

Methods used in the diagnosis of diseases must be accurate, fast, and have a low probability of obtaining incorrect results.^[29]^ Unlike the shortage of clinicians and expensive manual diagnosis in most countries, machine learning-based diagnosis can significantly improve the healthcare system and reduce misdiagnoses caused by clinicians’ stress, fatigue, and inexperience. Therefore, machine learning models can be used for the early diagnosis of diseases. Therefore, machine learning algorithms have attracted considerable attention in the medical health field and have been used to solve various medical problems. Obido et al used decision tree, logistic regression, support vector machine, random forest, adaptive boosting (AdaBoost), and extreme gradient boosting (XGBoost) algorithms for hepatitis B diagnosis, and achieved balanced accuracies of 75%, 82%, 75%, 86%, 92%, and 90%, respectively.^[30]^ In another study, a combination of an information gain-based feature selection technique and a cost-sensitive AdaBoost classifier was used to effectively detect chronic renal failure using the proposed cost-sensitive AdaBoost method trained with a reduced feature set and achieved the best classification performance with 99.8% accuracy, 100% sensitivity, and 99.8% specificity_._^[31]^ Ebiaredoh-Mienye et al achieved 98%, 97%, and 91% test accuracy for chronic kidney disease, cervical cancer, and heart disease, respectively, with machine learning models consisting of feature learning and classification stages integrating a sparse autoencoder and softmax regression.^[32]^ As we move towards individualized medicine, the use of machine learning to analyze patients’ data about their diseases will increase. As progress continues in idealized medical approaches, it is imperative to develop methods for optimal data analysis and the integration of multimodal data.^[33]^

In this study, we introduced a new dataset, including bioimpedance measurements, predominantly used by dieticians, and laboratory data showing patients’ metabolic markers, as well as personal information, such as age, sex, weight, and height, to predict gallstones. We used nine machine learning techniques to analyze the efficiency of the proposed features and preferred to use gradient boosting techniques to achieve the best results. The proposed technique efficiently detects gallstones as an alternative to ultrasonography.

## 2. Materials and methods

This was a prospective descriptive study based on data obtained from 454 participants treated at the Internal Medicine Outpatient Clinic of Ankara VM Medical Park Hospital between June 2022 and June 2023. This study was approved by the Ethics Committee of the Ankara City Hospital Medicine (E2-23-4632). After omitting the records of 134 individuals who had undergone gallbladder surgery, our refined dataset was narrowed down to 319 entries.

**Figure 1.**
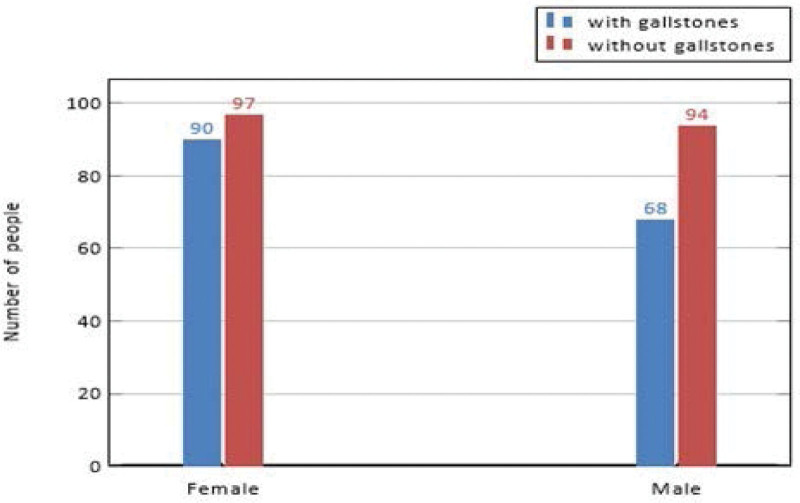
Gender-wise distribution of individuals with and without gallstones.

Of these, 161 individuals had gallstones and the remaining 158 were deemed healthy controls. The dataset covered the following 38 features: gallstone status, age, sex, comorbidities, hypertension, hypothyroidism, lean mass, protein content in the body, visceral adiposity index, bone mass, hyperlipidemia, diabetes mellitus, height, weight, body mass index (BMI), total body water (TBW), extracellular water (ECW), intracellular water, muscle mass, degree of obesity, total fat content, visceral fat area, visceral muscle area, hepatic fat accumulation, glucose levels, total cholesterol, low density lipoprotein (LDL), high density lipoprotein (HDL) cholesterol, triglycerides, aspartat aminotransferaz, Alanin aminotransferaz (ALT), alkalen fosfataz, creatinine, glomerular filtration rate (GFR), C-reactive protein (CRP), hemoglobin (HGB), and vitamin D concentration. In this section, we describe these features in detail. Gallstone status presents information on whether a person has gallstones or not. This feature is a binary variable, where 1 represents a person with gallstones; otherwise 0. The age feature represents the age of a person whose values change between 20-96 years. Sexual information was presented by gender, with values of 0 for males and 1 for females. A genderwise visualization is shown in Figure 1. The dataset included 187 females whose ages changed from 25 to 78 years, and 90 of the 157 patients had gallstones. Furthermore, the dataset included 162 males whose ages ranged from 20 to 96 years, and 68 of the 162 patients had gallstones.

The comorbidity feature is also a binary variable: if a person has comorbidities, the value of the comorbidities is 1; otherwise, it is 0. Hypertension, hypothyroidism, and hyperlipidemia were the binary features. If a person has an associated feature (whose value is 1) or not (whose value is 0), diabetes mellitus is also a binary variable. If a person has diabetes mellitus, the value of this feature is 1; otherwise 0. The height feature represents a person’s height (cm), and is measured barefoot. The weight feature represents a person’s weight (kg), and is measured with a person wearing thin clothing of approximating 0,1 kg. BMI is an indicator of obesity and was calculated as weight/height2 (kg/m2).

BIA was performed by all participants using the Tanita MC780 device, and the following features were determined: The total body water, extracellular water, and intracellular water were measured in kilograms. The total body fat ratio, lean mass, degree of obesity, and protein content of the body are presented as percentages (%). Bone mass, muscle mass, total fat content, visceral fat area, and visceral muscle area were measured in kilograms (kg).

Hepatic fat accumulation (fatty liver stage) was detected by ultrasonography. The following features were obtained from blood tests: glucose levels (mg/dL), total cholesterol (mg/dL), LDL cholesterol (mg/dL), HDL cholesterol (mg/dL), triglycerides (mg/dL), aspartat aminotransferaz (U/L), ALT (U/L), alkalen fosfataz (U/L), creatinine (mg/dL), GFR (ml/seconds), CRP level (mg/L), hemoglobin count (g/dL), and vitamin D concentration (ng/mL).

### 2.1. Machine learning methods

We used k-nearest neighbors, multilayer perceptron, support vector machines, decision tree, random forest, adaptive boosting, gradient boosting, naïve Bayes, and logistic regression techniques to predict whether a person had a gallstone based on bioimpedance and laboratory data. In the next section, we describe our proposed algorithm.

### 2.2. K-nearest neighbors (KNN)

KNN^[34–37]^ is a machine learning method used in classification problems. This method first calculates the similarity of the input dataset in its learning set, and classifies it according to predetermined threshold values. The performance of this method is determined by the number of nearest k neighbors in the learning set as well as the distance measure. A majority vote is assigned to the new data sample. The optimal determination of the distance measure and k value is critical. In this study, the optimum values of these hyperparameters were achieved through 10-fold cross-validation using grid search. In the grid search approach, the k value was chosen to be between 1 and 30, and the possible distance measures were assigned as Manhattan, Euclidean, or Chebyshev.

### 2.3. Multilayer perceptron (MLP)

The MLP^[38]^ is a type of feed forward artificial neural network. By increasing the number of input, hidden, and output layers, MLPs excel at deciphering nonlinear relationships by inserting one or more hidden layers between input and output layers. The number of neurons in these layers and the activation function used were improved to improve network performance. In this study, we optimized these parameters using 10-fold cross-validation coupled with a grid search, iterating over neuron counts such as 50, 100, 150, 250, 500, and 1000, and activation functions such as logistic sigmoid, hyperbolic tangent, and rectified linear units.

### 2.4. Support vector machines (SVM)

SVM^[39,40]^ is another successful learning algorithm that achieves a higher performance for classifying complex systems. The SVM classifies data points by constructing a hyperplane and minimizing errors in binary classification problems. The closest sample points are called the support vectors. The radial basis function (RBF) is an efficiently used kernel function for overcoming nonlinearity^[41,42]^ and we used the RBF kernel in this study. Selection of the RBF kernel parameter (γ) and penalty parameter (c) related to the SVM model is crucial. These parameters were determined by performing 10-fold cross-validation with a grid search. The values of the kernel and penalty parameters were changed in the set (2−5, 2−1, 29) and γ in (2−9, 2−5, 2−1, 23).

### 2.5. Decision tree (DT)

The primary principle of DT^[43]^ is to combine numerous simple decisions to achieve an ultimate decision. The performance of a DT classifier significantly depends on how the tree is built. Splitting approaches for decision trees include information gain and Gini index. The Gini index was used in this study. We also performed hyperparameter tuning using a grid search and used a similar strategy with^[44]^ parameters to determine the possible hyperparameter values (maximum tree depth: 5, 10, 15, 20, and 25; minimum samples per leaf: 1, 2, and 3; and minimum samples per split: 3, 4, 5, 6, and 7).

### 2.6. Random forest (RF)

RF^[43]^ is an ensemble approach for solving classification issues that combines several decision trees. The final choice is made by integrating the outcomes of a series of decision trees. Each tree was constructed individually and relied on a random vector selected from the dataset to ensure that all the trees had the same distribution. Random feature selection and bootstrap aggregation were used to average the predictions from all trees. Note that hyperparameter selection is identical to that of the DT approach.

### 2.7. Adaptive boosting

AdaBoost^[45]^ is a well-known boosting approach that combines numerous basic weak classifiers to form a powerful classifier. AdaBoost is also an iterative approach because it trains basic classifiers successively. In the first iteration, the weights of the training samples are assigned the same value (e.g., 1.0). The weights of the training samples were improved with respect to error rates. Higher weights were assigned to the training samples with higher error rates. Hyperparameter selection is an important procedure for boosting algorithms to address overfitting, because it decreases the bias error and builds robust predictive models. The AdaBoost technique has two hyperparameters: a learning rate with potential values of 1.0, 0.15, 0.1, 0.05, 0.01, 0.005, and 0.001 and a maximum number of estimators with possible parameters of 1.0, 0.15, 0.1, 0.05, 0.01, 0.005, and 0.001.

### 2.8. Gradient boosting classifier (GB)

Gradient Boosting is a strong machine learning technique that combines gradient descent with boosting. The gradient-boosting decision tree integrates several weak base classifiers into a powerful classifier. Unlike standard boosting algorithms, which weigh positive and negative samples, the GB classifier achieves global algorithm convergence by following the direction of a negative gradient. The main steps of the GB classifier are described in.^[46]^ Extreme gradient boosting is a regularized form of gradient boosting. It performs advanced regularization called L1 regularization (Lasso) and L2 regularization (Ridge), which improves the capabilities of the model generalization. XGBoost avoids overfitting better than gradient boosting via regularization.

### 2.9. Naïve Bayes (NB)

NB^[43]^ is a statistical model based on the Bayes’ theorem as shown in Equation 1, the NB classifier assumes that the influence of a given characteristic in the class is independent of other attributes. In this equation, *X* represents the feature set, and c represents the class set. Even if these characteristics are linked, they can be independently assessed. This premise simplifies the computation, and is naïve for this reason.

$$\begin{array}{l} P\left(c|X\right)=\frac{P\left(X|c\right)P\left(c\right)}{P\left(X\right)} \end{array}$$ (1)

where *P*(*c|X*) is the posterior probability, *P*(*X*|*c*) the likelihood, *P*(*c*) the class prior probability, and *P*(*X*) the predictor prior probability.

### 2.10. Logistic regression

Logistic regression (LR)^[43]^ is a machine learning technique that is mostly used for classification problems to predict the likelihood that an instance belongs to a specified class. Logistic regression was used as the classification method. This is called regression, because it takes the output of the linear regression function as the input and estimates the probability for a given class using the sigmoid function shown in Equations 2 and 3.

$$\begin{array}{l} z=w_{1}x_{1}+\;w_{2}x_{2}+\ldots +\;w_{n}x_{n}+b\;\; \end{array}$$ (2)

$$\begin{array}{l} \;sigmoid\;\left(z\right)={\left(1+e^{-z}\right)}^{-1} \end{array}$$ (3)

where *w*1, *w*2, …, *w*n are regression coefficients; *x*1, *x*2, … *x*n are features; and *b* is a constant. This method is suitable for binary classification and can be efficiently used in medicine.

### 2.11. Evaluation criteria

For an astute appraisal of the machine learning algorithms, we incorporated several standard metrics such as Precision, Recall, F-measure, and Accuracy. A succinct description of these metrics is presented in Table 1. True Positive corresponds to instances where gallstones were correctly identified as being present. False positives pertain to instances incorrectly flagged as having gallstones when, in reality, they are absent. True Negative designates instances correctly recognized as lacking gallstones. False Negative refers to situations in which the presence of gallstones went undetected, despite their actual presence. This comprehensive framework provides a holistic view of the predictive capabilities of the model in discerning the presence of gallstones. We also evaluated the results for the Area Under the Receiver Operating Characteristic (AUC-ROC) metric. The area under the curve (AUC) score changed from 0 to 1. The machine learning algorithm correctly predicted all the results when the AUC score was 1.

**Table 1:** Metrics for evaluating machine learning model performance.

Measure	Formula
Precision	$\frac{TP}{TP+FN}$
Recall	$\frac{TP}{TP+FN}$
F-measure	$\frac{2\;\;\;x\;\;\;Precision\;\;\;x\;\;\;Recall}{Precision+\;\;\;Recall}$
Accuracy (Acc)	$\frac{TP+TN}{FN+TP+TN+FP}$

FN = false negative, FP = false positives, TN = true Negative, TP = true positive.

## 3. Results

All computational tasks were executed on a machine equipped with an Intel Core i7 CPU clocked at 2.6 GHz, complemented by 16 GB of RAM, and operated in the Ubuntu 18.04.03 LTS operating environment. The machine learning algorithms were instantiated using Scikit-learn toolkit, a robust open-source software tailored to the Python environment. The dataset was divided into training and testing datasets. We used 70% of the dataset for training and remaining 30% of the dataset was used for testing.

Machine learning techniques have been employed to discern patterns from both bioimpedance and laboratory data for the identification of gallstones. In our quest for optimal results, we calibrated the parameters of these algorithms using grid-search methodologies. The performance assessment was anchored to the metrics described in previous sections.

**Figure 2.**
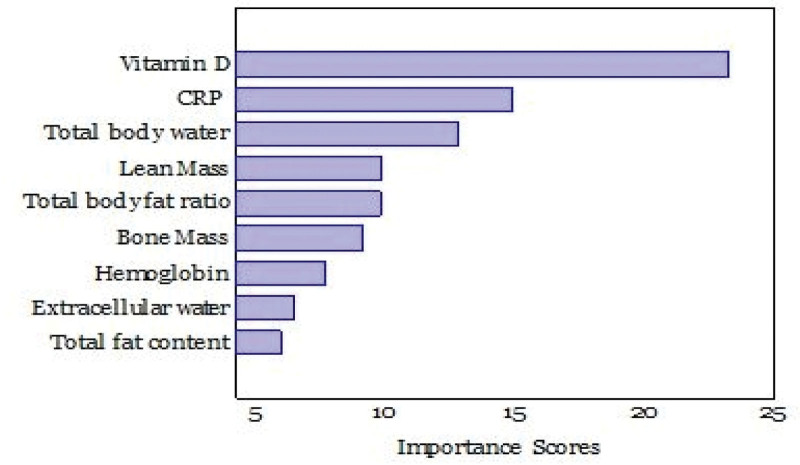
Feature importance scores are determined by F-score using ANOVA. CRP = C-reactive protein.

First, we evaluate the importance of this feature. Feature importance scores were derived using the Analysis of Variance technique, and the resultant data are tabulated in Table 2. Higher F-scores suggest that a feature has a greater discriminative power, meaning that it can distinguish between instances with gallstones more effectively. Hence, the top 32 features were selected for Analysis of Variance F-scores by eliminating features with F-scores below 0.70. As shown in Table 2, vitamin D exhibited the highest F-score (23.42), indicating that it might play a crucial role in predicting gallstones. CRP and total body water also had notable scores of 15.0 and 12.96, respectively, and these factors provided significant information for gallstone prediction. Lean masses, total body fat ratio, and bone masses are crucial features for gallstone detection. Nine significant features for gallstone prediction were identified, as shown in Figure 2.

**Table 2:** Feature importance based on ANOVA F-score.

Feature Name	F-Score	Feature Name	F-Score	Feature Name	F-Score
Vitamin D	23.42	AST	4.14	Hypothyroidism	1.12
CRP	15.0	Creatinine	4.01	Obesity	1.08
TBW	12.96	BMI	3.5	Triglyceride	1.0
Lean Mass	9.96	ICW	3.05	Weight	0.95
TBFR	9.93	ALP	2.99	Comorbidity	0.94
Bone Mass	9.24	Height	2.91	ALT	0.78
ECW	6.59	Hypertension	2.48	Hemoglobin	0.63
TFC	6.07	Muscle Mass	2.34	GFR	0.48
Hyperlipidemia	5.58	HFA	2.22	VAI	0.29
HDL	5.4	Protein	2.09	Total cholesterol	0.22
Gender	5.11	VMA	1.64	Glucose	0.17
VFA	4.41	LDL	1.15		

ALP = alkalen fosfataz, ALT = alanin aminotransferaz, AST = aspartat aminotransferaz, BMI = body mass index, CRP = C-reactive protein, ECW = extracellular water, GFR = Glomerular filtration rate, HDL = high density lipoprotein, HFA = hepatic fat accumulation, ICW = intracellular water, LDL = low density lipoprotein, TBFR = total body fat ratio, TBW = total body water, TFC = total fat content, VAI = visceral adiposity index, VAI = visceral adiposity index, VFA = visceral fat area, VMA = visceral muscle area.

HGB, HDL, BMI, height, and diabetes had moderate effects on gallstone detection, and these features still played a significant role in the prediction process. Furthermore, sex had a moderate significance in detecting gallstones. This suggests that there is a certain degree of difference between sexes in gallstone prediction. In contrast, ALT level, age, HGB level, GFR, visceral adiposity index, total cholesterol level, and glucose level had little effect on gallstone detection. Therefore, we excluded these features from the dataset. Table 2 presents a structured approach to understanding the relative significance of various features in gallstone prediction. Using this data, researchers and medical professionals can refine their prediction models and diagnostic processes to enhance accuracy and efficiency.

Next, we present the results of the machine learning techniques to evaluate whether the proposed features described in our dataset predict gallstones. The results for each classifier based on precision, recall, F-measure, and AUC scores are summarized in Table 3. First, we evaluated precision scores ranging from 0.52 to 0.93. The XGBoost model achieved a precision of 0.93, indicating its superior ability to identify positive instances correctly. Conversely, SVM demonstrated the lowest precision of 0.52. The RF and GB models achieve precision pinnacles of 0.91. MLP, LR, NB, DT, KNN, SVM, and AdaBoost achieved precision values of 0.76, 0.86, 0.61, 0.78, 0.53, 0.52, and 0.86, respectively. Second, we evaluated the recall values that change from 0.29 to 0.87. NB stood out, with the highest recall of 0.87, denoting its proficiency in capturing the majority of positive instances. SVM registers the lowest recall of 0.29. This underscores the potential false-negatives produced by the SVM model. RF, MLP, LR, NB, DT, KNN, GB, AdaBoost, and XGBoost achieved recall values of 0.81, 0.56, 0.83, 0.87, 0.62, 0.44, 0.81, 0.81, and 0.75, respectively. Next, we analyzed F-measure values, providing a harmonized evaluation of precision and recall that range from 0.37 to 0.86. The RF and GB classifiers excel in this regard, recording a maximum F-measure of 0.86. The SVM lagged in this metric by a value of 0.37, emphasizing a balanced gap between its precision and recall. Adaboost and XGBoost had the same F-measure value of 0.83. MLP, LR, NB, DT, KNN, and AdaBoost reached F-measure values of 0.64, 0.84, 0.71, 0.69, and 0.48, respectively. Finally, we evaluated the AUC scores, which ranges from 0.49 to 0.85. The AUC scores were similar to the accuracy values. The best results were obtained when the RF and GB were used. The MLP, LR, NB, DT, KNN, SVM, AdaBoost, and XGBoost achieved AUC scores of 0.67, 0.83, 0.60, 0.70, 0.49, 0.48, 0.82, and 0.84, respectively. In summary, the GB method exhibits commendable performance across the three metrics considered.

**Table 3:** Performance evaluation of machine learning classifiers on the proposed dataset.

Method	Precision	Recall	F-measure	AUC
RF	0.91	0.81	0.86	0.85
MLP	0.76	0.56	0.64	0.67
LR	0.86	0.83	0.84	0.83
NB	0.61	0.87	0.71	0.6
DT	0.78	0.62	0.69	0.7
KNN	0.53	0.44	0.48	0.49
SVM	0.52	0.29	0.37	0.48
GB	0.91	0.81	0.86	0.85
Adaboost	0.86	0.81	0.83	0.82
XGBoost	0.93	0.75	0.83	0.84

AdaBoost = adaptive boosting, DT = decision tree, GB = Gradient Boosting Classifier, KNN = K-Nearest Neighbors, LR = logistic regression, MLP = multilayer perceptron, NB = Naïve Bayes, RF = random forest, SVM = support vector machines, XGBoost = extreme gradient boosting.

**Figure 3.**
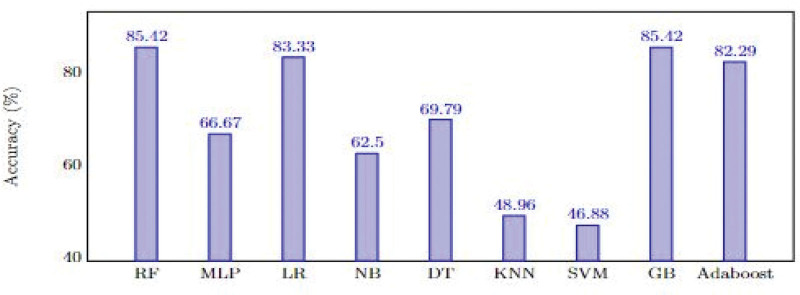
Comparative accuracy of the machine learning algorithms. AdaBoost = adaptive boosting, DT = decision tree, GB = Gradient Boosting Classifier, KNN = K-Nearest Neighbors, LR = logistic regression, MLP = multilayer perceptron, NB = Naïve Bayes, RF = random forest, SVM = support vector machines.

Furthermore, accuracy plays an essential role as a performance metric in discerning the effectiveness of machine learning classifiers. It quantifies how often a model predicts the correct output, and offers direct insight into the overall capability of an algorithm in a given context. Although multiple metrics exist to measure the performance of machine learning classifiers, accuracy offers a straightforward and comprehensible depiction of a model’s proficiency. Figure 3 delves deeper into the comparative assessment of the accuracy achieved by the various classifiers on the dataset. The accuracy values change from 46.88% to 85.42%; while GB and RF achieved the best performance, the performance of SVM was the lowest. LR and XGBoost had the same accuracy value of 83.33%. MLP, LR, NB, DT, KNN, SVM, and AdaBoost achieved accuracies of 66.67%, 83.33%, 62.5%, 69.79%, 48.96%, 46.88%, and 82.29%, respectively.

## 4. Discussion

Ultrasound imaging is the primary and effective diagnostic tool for gallstones. However, the interpretation of these images requires extensive expertise, particularly when symptoms overlap.^[47]^ Limitations in resources and shortages of skilled analysts in some regions emphasize the need for more efficient ultrasound interpretation methods, particularly in remote areas. Given BIA’s noninvasive nature of BIA and its potential ties with metabolic health, there is a burgeoning interest in leveraging this tool to forecast gallstone risk.^[26]^ Unlike traditional algorithms that rely on pre-established rules or equations, machine learning algorithms deploy computational techniques to derive insights directly from data. With the influx of data, these algorithms have improved their predictions, drawing parallel to the way humans learn from experience.

Our bioimpedance analysis and laboratory data showed that vitamin D level, CRP level, total body water, and fat-free mass are important features, and the gradient boosting technique achieved the highest accuracy of 85.42% in predicting gallstones. Considering that abdominal ultrasonographic imaging is a useful, noninvasive method for diagnosing gallstones and that this method has a sensitivity of 84% in detecting gallstones,^[15–17]^ our method can be considered quite effective. Salem et al^[33]^ identified gallstones from metabolites and their clinical features using machine learning methods. Their dataset included 218 samples from 100 gallstone patients. They used 50 features consisting of blood and clinical risk factors: age, sex, obesity, body mass index, hemoglobin, and cholesterol. They achieved an accuracy of 83% when the convolutional neural network was used. By contrast, the proposed method achieved nearly the same accuracy on a relatively large dataset. With fewer features. When the number of samples in the dataset used by Salem et al^[33]^ increased, the overall accuracy of their method decreased significantly. Yao et al^[48]^ used deep learning to extract the features from an imaging gallstone dataset. These features can be used to group different types of gallstones based on their chemical composition. Veena and Gowrishankar^[49]^ used deep learning methods to detect gallstones in 30 ultrasound images. Their method achieved a precision of 0.78, a recall of 0.98, and an F-score of 0.93. The main limitation of their method is the number of images and manual annotation of the images.

In our study, it has been shown that gallstone formation is more common in vitamin D deficiency and high levels of CRP. Cholesterol is a precursor of gallstone formation,^[50]^ 7-dehydrocholesterol is a common precursor of vitamin D, and cholesterol^[51]^ conversion of 7-dehydrocholesterol to cholesterol rather than vitamin D synthesis may increase gallstone formation. These findings suggested a relationship between vitamin D deficiency and gallstone formation. Another study found that vitamin D deficiency in pregnant women caused gallbladder stasis and reduced gallbladder ejection fraction.^[52]^ Bile stasis is one of the mechanisms by which vitamin D deficiency facilitates gallstone formation. In a study comparing patients with gallstones and healthy controls, high-sensitivity CRP levels were higher in patients with gallstones.^[53,54]^

In China, a cross-sectional study by Shabanzadeh et al showed that CRP level was positively associated with gallstones.^[21]^ Liu et al^[55]^ reported that CRP concentration was an independent risk factor for newly developing gallstones. The mechanisms underlying gallstone formation and CRP level remain unclear. Obesity plays an important role in the formation of gallstones. It is thought that obesity-related interleukin-6 secretion causes an increase in the CRP levels in hepatocytes.^[56,57]^ Berk et al^[58]^ found that chronic inflammation, for any reason, causes calcium accumulation in the gallbladder wall. These two mechanisms may explain the pathogenesis of CRP and stone formation.

When the literature was examined for gallstone formation with TBW measured by bioimpedance, Corradini et al^[59]^ reported in their study on the risk of gallstone formation of sulfate-bicarbonate-calcium water consumption that water consumption had a positive effect on the risk of gallstones. In the study by Bostan et al in which they examined the relationship between gallstones and total body fluid, no relationship was found between total body fluid ratio and the risk of gallstone formation.^[26]^ However, another study conducted on a group of patients with obstructive jaundice showed that the amount of TBW and ECW decreased.^[60]^ In our study, total body water (TBW) and ECW stood out as two important parameters in the model we proposed for gallstone prediction.

It has been proven that there is a relationship between gallstone formation, abnormal glucose metabolism, and hyperinsulinemia.^[61]^ In another study, gallstone formation was higher in patients with type-2 diabetes, but this could not be statistically proven.^[62]^ Everson et al found that individuals with hemoglobin metabolism disorders, such as sickle hemoglobinopathy, have an enlarged gallbladder that contributes to the formation of gallstones.^[63]^ A study by Fu et al^[64]^ also suggested that the ratio of beta-lipoprotein and LDL cholesterol to HDL cholesterol is a risk factor for gallbladder stone formation. In light of these studies, it is understood that there may be a relationship between hemoglobin levels and gallstones, potentially affected by factors such as glucose and lipid metabolism. Therefore, these parameters were included in the dataset in which we created the gallbladder stone prediction model, and the algorithm determined hemoglobin to be an important parameter in gallbladder stone prediction.

Increased intra-abdominal fat ratio, regional fat distribution imbalances in the body, and central adiposity indicated by the waist-hip circumference ratio increased gallbladder volume and the risk of gallstone formation in both men and women.^[28,65,66]^ As a result of our study, the total body fat ratio has emerged as an important parameter for the prediction of gallstones.

However, our study had some limitations. The demographic framework of our study population may not comprehensively capture global diversity. Therefore, extrapolating our results to a global scale requires rigorous validation across heterogeneous demographic cohorts. However, when integrating machine learning into clinical decision-making processes, continuous efforts are required to improve and validate the techniques to maintain their clinical relevance.

Consequently, this study showed the importance of data, including bioimpedance and laboratory characteristics, in detecting gallstones as a result of analysis using nine different machine learning methods. This not only highlights the presence of gallstones but also provides clinicians and healthcare professionals with avenues for potential early diagnosis and intervention based on these markers. Bioimpedance and laboratory assessments hold great promise, and can be used for disease prediction, similar to abdominal ultrasonography. Larger studies with different populations are required to generalize the results.

## Author contributions

**Conceptualization:** İrfan Esen, Selin Aktürk Esen.

**Data curation:** İrfan Esen, Hilal Arslan, Mervenur Gülşen, Nimet Kültekin, Oğuzhan Özdemir.

**Formal analysis:** İrfan Esen, Hilal Arslan, Selin Aktürk Esen.

**Investigation:** İrfan Esen, Mervenur Gülşen, Nimet Kültekin.

**Methodology:** İrfan Esen, Hilal Arslan.

**Project administration:** Hilal Arslan.

**Resources:** İrfan Esen, Oğuzhan Özdemir.

**Software:** Hilal Arslan, Selin Aktürk Esen.

**Supervision:** Selin Aktürk Esen, Mervenur Gülşen, Nimet Kültekin, Oğuzhan Özdemir.

**Validation:** Selin Aktürk Esen, Oğuzhan Özdemir.

**Writing – original draft:** İrfan Esen.

**Writing – review & editing:** İrfan Esen, Oğuzhan Özdemir.
